# HCV Viral Decline at Week 2 of Peg-IFN-Alpha-2a/RBV Therapy as a Predictive Tool for Tailoring Treatment in HIV/HCV Genotype 1 Co-Infected Patients

**DOI:** 10.1371/journal.pone.0099468

**Published:** 2014-06-19

**Authors:** Antonio Rivero-Juarez, Luis F. López-Cortés, Angela Camacho, Almudena Torres-Cornejo, Ana Gordon, Rosa Ruiz-Valderas, Julian Torre-Cisneros, Juan A. Pineda, Pompeyo Viciana, Antonio Rivero

**Affiliations:** 1 Unidad de Enfermedades Infecciosas, Instituto Maimonides de Investigación Biomédica de Córdoba (IMIBIC), Hospital Universitario Reina Sofía de Córdoba, Córdoba, Spain; 2 Unidad Clínica de Enfermedades Infecciosas, Microbiología y Medicina Preventiva, Instituto de Biomedicina de Sevilla (IBiS), Hospital Universitario Virgen del Rocío/CSIC/Universidad de Sevilla, Seville, Spain; 3 Unidad de Enfermedades Infecciosas y Microbiología Clínica, Instituto de Biomedicina de Sevilla (IBiS), Hospital Universitario de Valme, Seville, Spain; National Institute for Viral Disease Control and Prevention, CDC, China, China

## Abstract

**Background:**

Optimizing HCV genotype 1 therapy in terms of response prediction and tailoring treatment is undoubtedly the cornerstone of treating HIV co-infected patients in clinical practice. Accordingly, our aim was to analyze the predictive value of HCV viral decline for sustained virological response (SVR), measured at a time point as early as week 2 of therapy with pegylated interferon alpha-2a plus ribavirin (Peg-IFN/RBV).

**Methods:**

Previously untreated HIV/HCV genotype 1 co-infected patients were included in this study. The HCV RNA titer was measured at week 2 after starting treatment with Peg-IFN/RBV. The likelihood of reaching SVR when HCV RNA viral titers declined at week 2 was evaluated relative to predictive baseline factors.

**Results:**

A total of 192 HIV/HCV genotype-1 co-infected patients were enrolled in the study and began therapy. One hundred and sixty-three patients completed a full course of Peg-IFN/RBV treatment for 2 weeks and 59 of these (36.2%) reached SVR. An HCV RNA viral load decline of ≥1.5 log IU/mL at week 2 had the maximum positive predictive value for SVR (83.3%; 95% CI: 68.5%–92.9%) and was identified as the strongest independent predictive factor for reaching SVR across all baseline predictive factors.

**Conclusions:**

HCV viral decline at week 2 had a high predictive value for identifying patients with a high and low likelihood of reaching SVR using dual therapy, regardless of strong predictive baseline factors. This finding may be useful for developing a predictive tool to help tailor HCV genotype 1 therapy in HIV co-infected patients.

## Introduction

Adding a direct-acting antiviral (DAA) drug for the treatment of hepatitis C (HCV) to pegylated-interferon plus ribavirin therapy (Peg-IFN/RBV) substantially improves the sustained virological response (SVR) rate in HCV genotype 1 patients co-infected with the human immunodeficiency virus (HIV) [1]. HCV DAAs are currently available in routine clinical practice. The results obtained from using these new drugs in previously untreated co-infected HIV/HCV genotype 1 patients show significantly enhanced SVR rates in this subset of patients [1]. Nevertheless, there are several limitations to the addition of these drugs that could limit their use in those patients who are starting their first anti-HCV treatment. Perhaps the most important limitation worldwide is the substantial increase in therapy costs [2]. Therefore, even though DAAs increase the chances of reaching SVR, the use of Peg-IFN/RBV alone in HCV genotype 1 patients may remain the first-line therapeutic option in most countries according to World Health Organization (WHO) HCV treatment guidelines [1]. So, optimizing HCV genotype 1 therapy, in terms of response prediction and tailoring the treatment, is unquestionably the cornerstone for treating HIV co-infected patients in clinical practice.

Host genetic patterns are excellent clinical tools for estimating patient treatment outcomes after a full course of therapy with Peg-IFN/RBV [3]–[7]. However, achieving on-treatment virological milestones is the critical factor for determining outcome across all baseline factors [8]–[11]. Those patients who achieve a rapid virological response (RVR) even if they harbor theoretically unfavorable baseline factors have a high chance of achieving SVR [8], [9].

Bringing forward the predictive treatment time points could be an important aspect of clinical practice for providing each patient with the best treatment regimen earlier. The aim of this study therefore was to analyze the predictive value of HCV viral decline for SVR, as measured at the early time point of week 2 of therapy with Peg-IFN/RBV, relative to well-known predictive factors.

## Materials and Methods

### Selection of Patients

HIV-infected patients with previously untreated chronic hepatitis C caused by viral genotype 1 were included in the study. Those patients who were positive for the hepatitis B surface antigen (HBsAg) were excluded. Patients were prospectively followed-up in two reference hospitals in southern Spain. The criteria used to determine hepatitis C therapy followed the American Association for the Study of Liver Diseases (AASLD) guidelines [12]. Host, clinical and virological characteristics were collected. The liver fibrosis stage was determined by liver biopsy using METAVIR fibrosis scores. Transient liver elastography (FibroScan, Echosens, Paris) was used to measure the liver stiffness values of those patients who had not undergone a pre-treatment liver biopsy. Liver stiffness measurements were taken by a single experienced operator at each participating hospital, following a routine described elsewhere. Advanced liver fibrosis was defined as a liver fibrosis stage of F3 or higher in patients who had undergone a pre-treatment liver biopsy or as a baseline liver stiffness value of ≥11kPa [13]. IL28B genotyping assays are described elsewhere [3].

### Treatment Regimens, Virological Evaluation and Outcomes

All individuals were treated with Peg-IFN-alpha-2a at doses of 180 µg per week, in combination with a weight-adjusted dose of oral ribavirin (1000 mg/day for patients <75 kg and 1200 mg/day for patients ≥75 kg) for 48 or 72 weeks, following AASLD guidelines [12].

Plasma HCV RNA titers were measured using a quantitative RT-PCR assay (Cobas Taq Man, Roche Diagnostic Systems Inc., Pleasanton, CA, USA), with a detection limit set at 15 IU/mL. In addition to routine treatment point measurements of plasma HCV RNA, for the purpose of this study, HCV RNA titers were measured 2 weeks after starting treatment.

HCV genotypes were determined using a hybridization assay (INNO-LiPa HCV, Bayer Corp., Tarrytown, NY, USA). HCV genotype 1was defined as 1a or 1b.

RVR was defined as a serum HCV RNA titer below the 15 IU/mL detection limit at day 28 after start of treatment. Patients with HCV RNA viral decline lower than 2 log UI/mL at week 12 after starting treatment were classified as null responders (NRs). An end-of-treatment response (ETR) was defined as a serum HCV RNA concentration below the 15 IU/mL detection limit upon the completion of therapy. SVR was defined as a serum HCV RNA titer below the 15 IU/mL detection limit 24 weeks after completion of therapy. Viral relapse (REL) was defined as detectable serum HCV RNA after having reached ETR.

### Statistical Analyses

Continuous variables were expressed as the mean ± standard deviation and were analyzed using the Student’s *t*-test, the Mann-Whitney *U*-test or the Kruskal-Wallis test. Categorical variables were expressed as number of cases (percentage). Frequencies were compared using the χ^2^ test or Fisher’s exact test. Significance was defined as a *p*-value of less than 0.05. For the purpose of this study, analyses were assessed using an on-treatment approach, excluding those who voluntarily dropped out or discontinued therapy due to adverse events. Patients were stratified according to the magnitude of HCV RNA viral decline 2 weeks after starting therapy. The likelihood of reaching SVR based on HCV RNA viral decline at week 2 was evaluated. Positive predictive value (PPV) was defined as the probability of achieving SVR based on HCV RNA reduction at week 2. For the PPV, a two-sided 95% confidence interval (95% CI) was calculated using the exact binomial distribution. Predictive accuracy was assessed by comparing area under the receiver operating characteristic (AUROC) curves. Variables associated with SVR were entered into logistic regression models. The analysis was carried out using the SPSS statistical software package, version 18.0 (IBM Corporation, Somers, NY, USA).

### Ethical Statement

The study was designed and performed according to the Helsinki Declaration and approved by the ethics committee of the Reina Sofía University Hospital, Cordoba, Spain. All patients provided a written informed consent form before participating in the study and gave permission for biological samples from routine diagnostic processes to be stored and processed in the Biobanco del Hospital Universitario Reina Sofía de Córdoba [biobank of the University Hospital Reina Sofía, Cordoba] (ISCIII reference: B.0000419), which forms part of the Biobanco del Sistema Sanitario Público de Andalucía [biobank of the Public Health System of Andalusia]. The CEIC [Clinical Trial and Ethical Committee] of the Hospital Universitario Reina Sofía de Córdoba approved the study protocol.

## Results

### Study Population

A total of 192 HIV/HCV genotype 1 co-infected patients started therapy and were enrolled in the study. Twenty-nine patients (15.1%) withdrew from therapy: 12 (6.2%) voluntarily dropped out and 17 (8.8%) discontinued therapy due to adverse events. Consequently, the on-treatment population that completed a full-course of therapy consisted of 163 patients. The most relevant demographic, virological and clinical characteristics of the on-treatment population are shown in Table 1.

**Table 1:** Demographic, virological and clinical characteristics of the on-treatment population.

Characteristic	On-treatment approach (N = 163)
Gender male, n (%)	133 (81.6)
Age (years), mean (SD)	41.5 (5.9)
BMI (kg/m^2^), mean (SD)	24.1 (4.2)
Previous AIDS-defining criteria, n (%)*	46 (28.2)
IDU, n (%)	132 (80.9)
Nadir CD4 cell count (cells/mL), mean (SD)	167 (132)
Baseline CD4 cell count (cells/mL), mean (SD)	554 (273)
HAART use, n (%)	150 (92.02)
Unquantifiable HIV RNA viral load, n (%)†	138 (84.6)
HCV genotype, n (%)	
** **1a	89 (54.6)
** **1b	62 (38)
** **1a/1b	6 (3.7)
** **Not genotyped	6 (3.7)
Liver fibrosis stage F3–F4, n (%)‡	83 (50.9)
ALT (IU/L), mean (SD)	79.03 (57.7)
LDL cholesterol (mg/dL), mean (SD)	84.6 (31.3)
IL28B genotype, n (%)§	
** **TT	11 (7.4)
** **CT	84 (56.7)
** **CC	53 (35.9)

**Legend:** number of cases (N); standard deviation (SD); Body mass index (BMI); kilograms (kg); meters (m); acquired immunodeficiency syndrome (AIDS); injecting drugs user (IDU); highly active antiretroviral treatment (HAART); human immunodeficiency virus (HIV); hepatitis C virus (HCV); alanine aminotransferase (ALT); low-density lipoprotein (LDL); interleukin 28B (IL28B); cytosine (C); thymine (T).

*Classified on the basis of Center for Disease Control and Prevention (CDC) recommendations *(Revised surveillance case definitions for HIV infection among adults, adolescents, and children aged <18 years and for HIV infection and AIDS among children aged 18 months to <13 years-United States, 2008. MMWR 2008; 57 (No RR-10): 1–14)*.

† HIV viral load was measured by PCR (CobasTaqMan, Roche Diagnostic Systems Inc., Pleasanton, CA, USA), detection limit set at 20 IU/mL.

‡ Fibrosis stage was determined by liver biopsy according to METAVIR fibrosis score staging or using liver transient elastography (FibroScan, Echosens, Paris).

§ Available in 148 (90.8%) patients.

### Virological Response

The overall SVR was 30.7% (95% CI: 24.5%–37.5%). Among the 163 on-treatment patients, 59 reached SVR (36.2%; 95% CI: 29.1%–43.8%). Sixteen patients (9.8%; 95% CI: 5.9%–15.1%) experienced REL. Fifty-two patients (31.9%; CI 95%: 25.1%–39.3%) dropped below 2 log IU/mL at week 12 of treatment and were classified as NRs.

Eighteen patients (11.04%; 95% CI: 6.9%–16.5%) achieved RVR, and all but 1 of these achieved SVR (PPV 94.4%; 95% CI: 75.5%–99.7%). Seven patients (4.3%; CI 95%: 1.9%–8.6%) reached unquantifiable HCV RNA at week 2, and all of these patients reached SVR (PPV 100%; 95% CI: 65.2%–100%).

### SVR Rate According to HCV RNA Viral Decline during the First Two Weeks of Therapy

The AUROC curve value for HCV RNA decline at week 2 for SVR was 0.788 (0.703–0.869). When patients were sorted according to HCV RNA viral decline at week 2, 36 patients (22.09%; 95% CI: 16.2%–28.9%) experienced a ≥1.5 log IU/mL decline. Of these, 30 achieved SVR (PPV 83.3%; 95% CI: 68.5%–92.9%) (Table 2).

**Table 2:** Positive predictive value of HCV RNA viral decline at week 2 after starting therapy for sustained virological response (on-treatment approach, N = 163).

HCV viral decline at week 2	N	SVR	PPV (%)	95% CI
≥0.25 log IU/mL	123	54	43.9	35.3–52.7
≥0.5 log IU/mL	87	47	54.02	43.5–64.3
≥1 log IU/mL	56	39	69.6	56.7–80.6
≥1.5 log IU/mL	36	30	83.3	68.5–92.9
≥2 log IU/mL	25	21	84	65.7–94.7
≥2.5 log IU/mL	21	17	80.9	60.2–93.6
≥3 log IU/mL	13	10	76.9	49.1–93.7

**Legend:** Hepatitis C virus (HCV); number of patients (N); Sustained Virological Response (SVR); Positive predictive value (PPV), sustained virological response (SVR); 95% confidence interval (95% CI), international unit per milliliter (IU/mL).

### PPV for SVR of HCV RNA Viral Decline at Week 2 According to Baseline Factors

**Figure 1:**
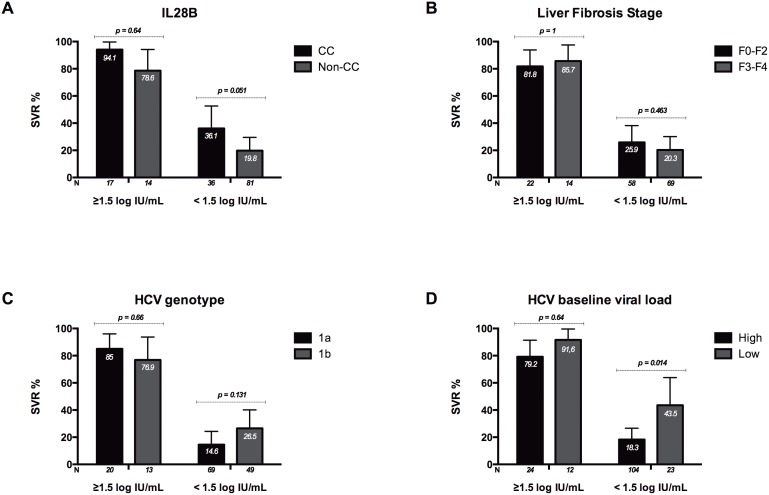
Comparison of Positive Predictive Value (PPV) for Sustained Virological Response (SVR) of viral decline of ≥ or < than 1.5 log IU/mL at week 2 after start of therapy relative to IL28B genotype (CC *versus* non-CC) (Figure 1A), liver fibrosis stage (F0–F2 *versus* F3–F4) (Figure 1B), HCV genotype (1a *versus* 1b) (Figure 1C), and baseline HCV viral load (≥600,000 IU/mL *versus* <600,000 IU/mL) (Figure 1D). *P* value was obtained using Fisher’s exact test or the chi-square test.

The PPV for SVR of an HCV RNA viral decline of ≥1.5 log IU/mL at week 2 was analyzed relative to IL28B genotype (Figure 1A), liver fibrosis stage (Figure 1B), HCV genotype (Figure 1C), and baseline HCV RNA viral load (Figure 1D). Among those patients who reached an HCV viral decline of ≥1.5 log IU/mL at week 2, no differences in SVR rates were found across all the baseline predictive factors analyzed (Figure 1).

**Figure 2:**
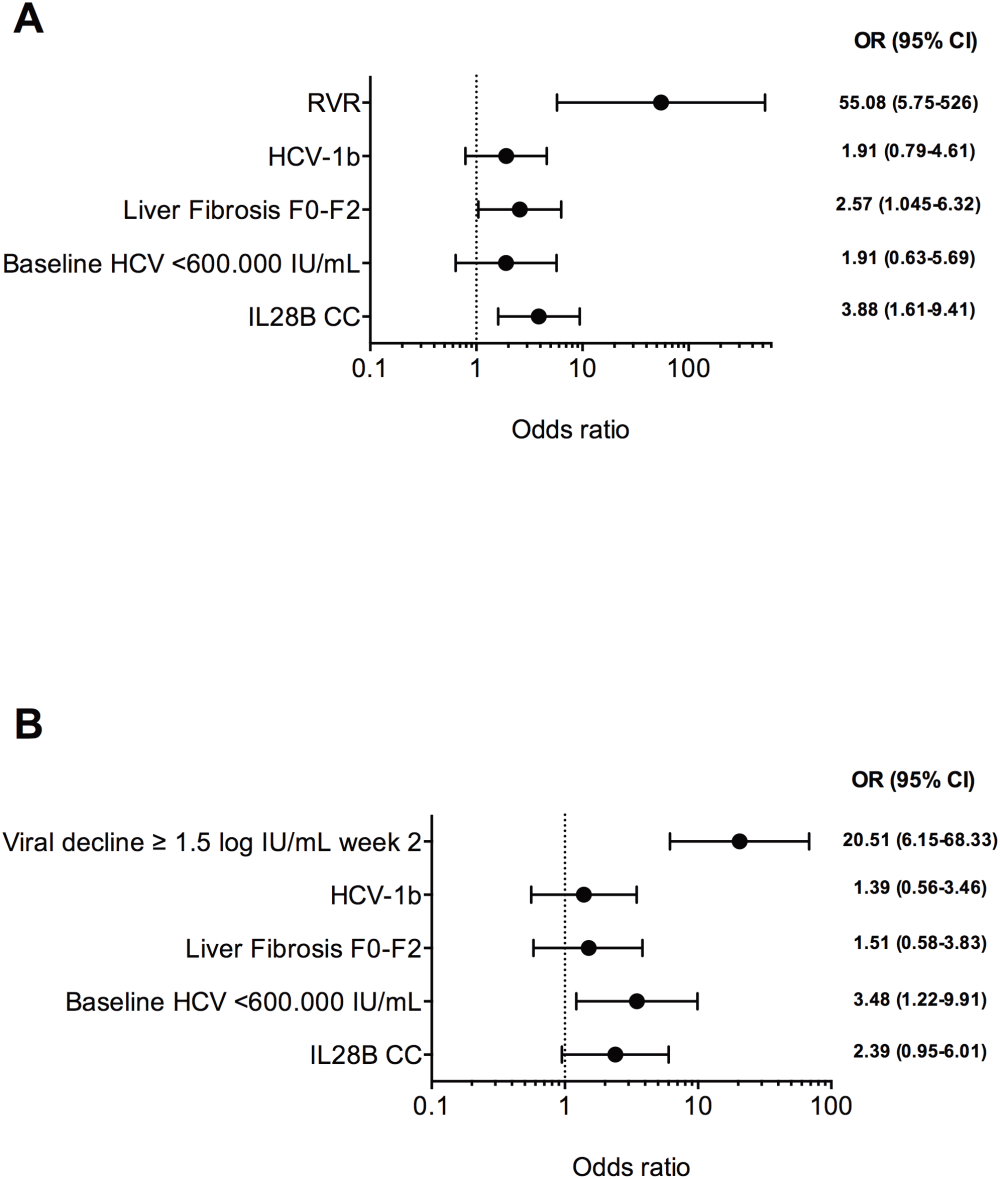
Multivariate logistic regression analysis for sustained virological response (SVR) including rapid virological response (RVR) (2A) and HCV viral decline of ≥1.5 log IU/mL at week 2 (2B). Odds Ratios (OR) and 95% Confidence Interval (95% CI) of each variable are shown to the right of the figure.

Two multiple logistic regression analyses for SVR were performed (Figure 2). Both RVR (Figure 2A) and HCV viral decline of ≥1.5 log IU/mL at week 2 (Figure 2B) were identified as the strongest independent predictive factors for reaching SVR in both models, independent of baseline predictive factors.

Table 3 shows the PPV for SVR of HCV viral decline at week 2, baseline HCV RNA viral loads and IL28B genotypes. The combination of these three factors had a PPV for SVR of 100% (95% CI: 60.7%–100%), whereas in patients who lacked these three predictive factors, the SVR rate was 15.1% (95% CI: 7.9%–25.3%). Furthermore, in patients carrying the IL28B CC genotype and having a low baseline HCV viral load, the chance of achieving SVR varied significantly according to whether they reached an HCV viral decline of ≥1.5 log IU/mL by week 2 (100%; 95% CI: 60.7%–100%) or not (50%; 95% CI: 18.4%–81.6%) (p = 0.085) (Table 3). Similarly, in patients carrying the unfavorable IL28B genotype and having higher baseline HCV viral loads, the likelihood of reaching SVR varied according to whether HCV viral decline of ≥1.5 log IU/mL was reached (77.8%; 95% CI: 43.8%–96.1%) or not (15.1%; 95% CI: 7.9%–25.3%) (p<0.001) (Table 3).

**Table 3:** Positive predictive value of HCV RNA viral decline at week 2 after starting therapy, HCV baseline viral load and IL28B genotype for Sustained Virological Response.

HCV decline ≥1.5 log IU/mL	Viral load <600,000 IU/mL	IL28B CC	N*	SVR	PPV (%)	95% CI
**+**	**+**	**+**	6	6	100	60.7–100
**+**	**–**	**+**	11	10	90.9	62.6–99.5
**+**	**+**	**–**	5	4	80	33.4–99
**+**	**–**	**–**	9	7	77.8	43.8–96.1
**–**	**+**	**+**	8	4	50	18.4–81.6
**–**	**+**	**–**	15	6	40	18.1–65.4
**–**	**–**	**+**	28	9	32.1	16.9–50.8
**–**	**–**	**–**	66	10	15.1	7.9–25.3

**Legend:** Hepatitis C virus (HCV); international unit per milliliter (IU/mL); Interleukin 28B (IL28B); number of patients (N); Sustained Virological Response (SVR); Positive predictive value (PPV); 95% Confidence Interval (95% CI); Matched condition (+); Unmatched condition (–). The negative condition refers to: HCV decline of <1.5 log IU/mL, baseline viral load ≥600,000 IU/mL, and IL28B Non-CC genotype.

*Total population included = 148.

## Discussion

Our study shows that HCV viral decline at week 2 of treatment exhibits a high PPV for SVR using Peg-IFN/RBV therapy. This finding could have important clinical applications since this strategy could enable the clinician to identify earlier those patients with a high likelihood of reaching SVR using dual therapy, with undoubted cost savings.

The use of HCV viral decline at week 4 of therapy for optimizing HCV treatment has been widely described [8], [12]. With respect to previously untreated HCV genotype 1 patients, achievement of RVR is the best treatment time point [8]. This has also been demonstrated in the DAA scenario [14]. In this context, patients on Peg-IFN/RBV therapy in a clinical trial who reached RVR had the same probability of achieving SVR as patients with Peg-IFN/RBV plus boceprevir [15]. Accordingly, the authors suggested that all patients initiating treatment against HCV genotype 1 should start therapy with two drugs, then use HCV viral kinetics to determine whether a third drug should be added during week 4 of therapy [15]. Nevertheless, even though RVR was the strongest predictive factor for SVR, only a small proportion of patients actually reached it, so limiting the benefit of the strategy [9], [10]. By increasing the number of classified patients and maintaining the PPV, evaluating the magnitude of HCV viral decline at week 4 considerably improved the clinical value, relative to the single criterion of RVR [9]–[11]. A recent study performed by our group described how including an evaluation of HCV viral decline at week 4 (≥2.5 log IU/mL) identified a higher number of potential SVR patients than the single criterion of RVR [9].

Our study shows that it is possible to predict a successful treatment outcome as early as 2 weeks after starting therapy. Based on our results, previously untreated HIV/HCV genotype 1 patients who experience an HCV viral decline of ≥1.5 log IU/mL at week 2 have a high chance of achieving SVR with a treatment based on Peg-IFN/RBV, and so allow us to identify a higher proportion of patients than when a prediction of RVR is used (22.1% and 11.04%, respectively). This suggests that the applicability of week 4 in predicting a successful treatment outcome could be brought forward to week 2 after start of therapy. Accordingly, clinicians could use an initial treatment phase of just 2 weeks with Peg-IFN/RBV, after which, depending on HCV viral decline, the treatment strategy could be personalized on a cost/benefit basis.

Our data provided one interesting finding concerning the clinical value of predicting the success of Peg-IFN/RBV therapy in co-infected HIV/HCV genotype1 patients. Among patients carrying both favorable baseline predictive factors (namely, the IL28B CC genotype and a low baseline HCV viral load), the likelihood of achieving SVR varied strongly according to viral decline at week 2 (Table 3). Similarly, among patients harboring both unfavorable factors (namely, the Il28B non-CC genotype and a high baseline HCV viral load), the likelihood of achieving SVR varied according to whether viral decline was higher or lower than 1.5 log IU/mL. Consequently, applying a treatment strategy to a therapy whose success is based only on baseline host factors has limited power for accurately predicting SVR because the early response to therapy matters more for prognosis than baseline host factors. This finding is consistent with a recent study published by our group, in which the likelihood of IL28 CC patients with a slow HCV viral decline at week 4 of treatment of reaching a successful treatment outcome using dual therapy was small [16]. Therefore, regardless of the patient’s baseline “prognosis profile”, the strategy of an initial 2-week phase of treatment with dual therapy might be an excellent tool for identifying those with a high chance of achieving SVR.

However, our study has several drawbacks. First, the number of patients was relatively small, which may have prevented us from detecting differences between baseline groups according to HCV viral decline at week 2. Second, the impact of using our findings on HIV/HCV genotype 1 patients is unknown, because we did not carry out a study of response-guided therapy. Third, we determined the IL28B genotype in 90.8% of patients; however, because it was not available for the entire study population, the impact of this host factor on statistical analysis may be limited. Finally, our results may not be generalizable to types of pegylated interferon preparation other than alpha-2a, although differences in treatment response and HCV viral decline using, for example, peg-interferon alpha-2b, would not be expected.

In conclusion, our findings show that HCV viral decline at week 2 after start of therapy could be used to predict the treatment outcome of Peg-IFN/RBV in HIV/HCV genotype 1 co-infected patients. The cut-off value of 1.5 log IU/mL yielded a high predictive value for identifying patients with a high and low likelihood of reaching SVR using dual therapy, regardless of whether strong predictive baseline factors were present. This finding may be useful for developing a predictive tool that may help in tailoring HCV genotype 1 therapy in HIV co-infected patients, so avoiding unnecessary costs and without compromising the chance of achieving SVR. Before applying our results to clinical practice, a response-guided therapy study is mandatory.
